# Color-thermal multispectral camouflage with VO_2_-based dynamic regulator

**DOI:** 10.1038/s41377-025-01968-x

**Published:** 2025-09-10

**Authors:** Chengcong Li, Cuicui Cao, Zhongshao Li, Zewei Shao, Fei Cao, Genshui Wang, Ping Jin, Hongjie Luo, Xun Cao

**Affiliations:** 1 State Key Laboratory of High Performance Ceramics and Superfine Microstructure, Shanghai Institute of Ceramics, Chinese Academy of Sciences, Shanghai, 200050 China; 2 Center of Materials Science and Optoelectronics Engineering, University of Chinese Academy of Sciences, Beijing, 100049 China; 3 The Key Lab of Inorganic Functional Materials and Devices, Shanghai Institute of Ceramics, Chinese Academy of Sciences, Shanghai, 200050 China; 4 Institute for the Conservation of Cultural Heritage, Shanghai University, Shanghai, 200444 China

**Keywords:** Mid-infrared photonics, Optoelectronic devices and components

## Abstract

Camouflage technology has garnered increasing attention for various applications. With the continuous advancement of detection technologies and the increasing variability of camouflage scenarios, the demand for multispectral dynamic camouflage has been steadily growing. In this work, we present a multispectral dynamic regulator based on phase-changing material vanadium dioxide (VO_2_) that can be dynamically and functional-independently regulated for reflective color and thermal radiation. It has been shown that the device can achieve a wide color gamut variation in visible band while simultaneously achieving highest emissivity tunability (Δ*ε*=-0.58) in the atmospheric window up to now, achieves multispectral camouflage spanning the visible and infrared spectra among VO_2_-based devices. To go a step further, we advance the device featuring long-term cycling stability to achieve thermal-electric dual-mode response and flexibility for a series real-world camouflage performance evaluation. We have also demonstrated the digital camouflage based on multispectral dynamic regulator through Neighboring Color Block Camouflage Algorithm, highlighting its potential for practical implementation in different camouflage scenarios. The device achieves multispectral dynamic camouflage, opening a path for advancing the technology development in both the scientific field and practical applications.

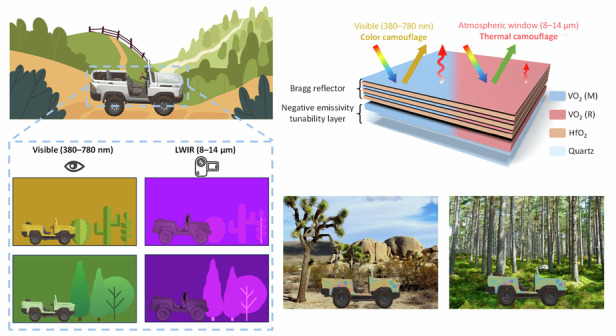

## Abstract (web summary)

A VO_2_-based dynamic regulator achieves color-thermal multispectral camouflage spanning the visible and infrared spectra.

## Introduction

Camouflage technology, by effectively blending into the environment, can significantly reduce target detectability, thereby enhancing survivability and mission concealment^1,2^. With the continuous advancement of detection technologies, traditional camouflage methods face challenges, leading to the emergence of innovative camouflage materials and multispectral camouflage technologies^3–7^. These technologies not only provide concealment within the visible spectrum but also offer effective camouflage across various spectra, including mid-infrared (MIR) thermal imaging, thereby achieving comprehensive stealth effects. However, static camouflage is prone to failure, especially when the target is exposed to dynamically changing environments and weather conditions^8–10^. For instance, when a vehicle transitions from a desert to a forest, static camouflage may become ineffective due to disparities between the object and its background in the visible and infrared spectra. Therefore, multispectral dynamic camouflage technology not only enhances the reliability of target concealment in dynamic and complex environments but also provides an effective solution to counter increasingly sophisticated detection technologies^11^.

To realize dynamic camouflage in these bands, materials whose optical properties can be regulated by thermal^9,12–14^, electrical^15–18^, strain^19,20^ or light^21–23^ is widely research. Among them, electrochromic material systems have been deeply explored for adaptive camouflage. As actively controlled materials with enhanced intelligence and tunability for camouflage technologies, their ability to switch within seconds represents a distinct advantage for achieving rapid and adaptable optical performance, highlighting their strong potential for practical applications. However, several limitations still exist in electrochromic devices to enhance the effectiveness of camouflage^24–33^ (Supplementary Table 1). Firstly, the optical tunability is limited by specific device structural factors, including high electrical conductivity and excellent infrared transparency electrodes, as well as the choice of encapsulation materials and methods, which can significantly affect the overall performance of the device. Then as for the multi-layer structure and complex design of electrochromic devices, stress and deformation may occur during flexible bending, affecting the ion migration efficiency between layers. And the device’s cycle life is also worth noting, which can be influenced by chemical changes in the electrochromic materials, hindered ion migration, electrode degradation, and electrolyte deterioration during repeated use. The implementation of multispectral dynamic camouflage requires not only materials with broadband modulation capability and tunability, but also sufficient flexibility and long cycle life for practical applications.

Vanadium dioxide (VO_2_) has emerged as a promising candidate for multispectral dynamic camouflage due to its marked variation in optical properties across visible and mid-infrared spectra before and after metal-insulator transition (MIT)^34,35^, which provides a material foundation for remarkable camouflage performance. Besides, elemental doping offers a pivotal strategy for tailoring the performance of VO_2_-based devices (phase transition temperature, transition width, spectral tunability, etc.)^36^, thereby significantly improving camouflage effectiveness. Moreover, its thermochromic properties enable the device to integrate both passive (temperature-adaptive) and active control (Joule heating) features^37,38^, making the dual-mode responsive device ideally suited for flexible control in complex environments. Nevertheless, despite VO_2_’s numerous outstanding properties mentioned above, there are still several key points in achieving multispectral dynamic camouflage that need to be emphasized. (1) According to the Stefan-Boltzmann law, the thermal radiation power can be expressed as *P* = *εσT*^4^, where *σ* is Stefan-Boltzmann constant, and the thermal radiation power (*P*) is proportional to emissivity (*ε*) of the object and the surface temperature (*T*) to the fourth power. Thus, the VO_2_-based device should have a superior negative emissivity tunability (Δ*ε* *=* *ε*_HT_ − *ε*_LT_ < 0, HT, LT represent high temperature and low temperature respectively) as temperature rises^39^, thereby counteracting the corresponding increase in thermal radiation power. (2) How to achieve both color and thermal camouflage in a single device. So far, the device structure based on VO_2_ to manipulate structural colors mainly consists of a Fabry–Perot multi-layer film composed of metal/VO_2_/metal^40–42^. The presence of IR-reflective metal layer in the structure will weaken the device’s overall mid-infrared emissivity tunability (Supplementary Table 2). Ensuring that the visible and infrared camouflage regulation do not interfere with each other, while maintaining optimal performance, requires a specialized structural design. (3) Due to the temperature limitations in the preparation of VO_2_ (typically above 300 °C)^13,43^, the device substrate is often rigid to withstand certain temperatures (e.g., quartz, silicon or sapphire). However, the device should be flexible enough for covering various target geometries, which will further enhance the adaptability and applicability of camouflage technology. Given these challenges, the advancement of a color-thermal camouflage system capable of functioning dynamically across multispectral continues to pose difficulties.

**Fig. 1:**
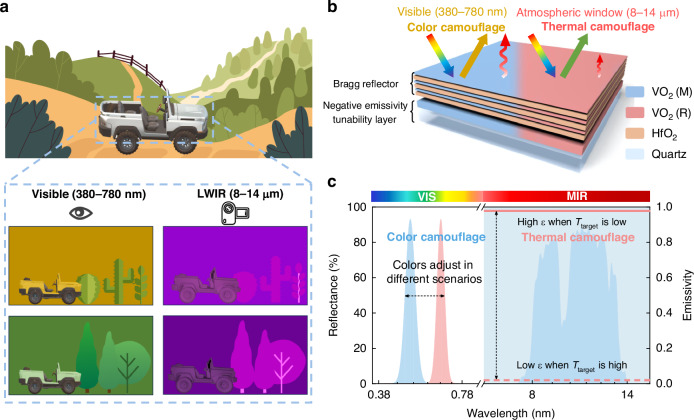
Schematic of multispectral dynamic regulator (MSDR) and its functioning principle. **a** A conceptual diagram of color-thermal camouflage. The MSDR-covered target can change its color to adapt to different scenarios (e.g., desert, forest, ocean) to achieve color camouflage. While the thermal radiation of the target is always consistent with background to avoid detection by infrared cameras, thus achieving thermal camouflage. The background illustrations are downloaded from website (https://www.islide.cc/) with permission under a Creative Commons license CC0 1.0. **b** Designed structure of MSDR composed of thermochromic vanadium dioxide (VO_2_), visible-infrared hafnium dioxide (HfO_2_) and quartz substrate. As the temperature varies, the device will change its reflective color and thermal emission. **c** Ideal spectrum for color-thermal camouflage under different device states. The colors should be adjusted in different scenarios to achieve color camouflage. The emissivity should decrease with the target temperature increases to achieve thermal camouflage. The deep-blue area indicates the transparent infrared atmospheric window (US standard 1976)

In this work, we present a multispectral dynamic regulator based on VO_2_, designed for tunable control in visible and mid-infrared bands. Leveraging the structural design of Bragg reflector and negative emissivity tunability layer, the VO_2_-based device ensures wide color gamut variation while simultaneously achieving highest negative emissivity tunability (Δ*ε*_8–14 μm_ = −0.58) to date, which experimentally achieves dynamic color-thermal camouflage among VO_2_-based devices (Supplementary Table 3). Most importantly, the color change and thermal emission regulation do not interfere with each other, realizing functionally independent control of visible and mid-infrared bands (Fig. 1a). Furthermore, by advancing the long-cycle stability device to achieve cooperative active-passive response and flexibility, and through simulation scenarios, we evaluate the camouflage performance of multispectral dynamic regulator in complex real-world environments. Our device provides opportunities for multispectral electromagnetic waves manipulation, paving the way towards advanced camouflage technology^4,17,20,44^, information anti-counterfeiting^45–47^, and thermal management^48,49^.

## Results

### Design of the multispectral dynamic regulator

The multispectral dynamic regulator (MSDR) is composed of two main functional components: the top thermochromic Bragg reflector, and the bottom negative emissivity tunability layer. These multilayer films, consisting of thermochromic vanadium dioxide (VO_2_) and visible-infrared transparent dielectric hafnium dioxide (HfO_2_), are alternately grown on high emissivity quartz with magnetron sputtering method (Fig. 1b). The significant variation in the optical constants of VO_2_ across a broadband will profoundly impact the overall optical performance of the device. In the visible band, the top Bragg reflector allows the device to dynamically modulate its reflective color under different temperature. Furthermore, by adjusting the film thicknesses, the central wavelength for reflection can be regulated correspondingly, providing a diverse range of reflective color combinations to align with various scenarios. As for the mid-infrared band, emissivity is modulated by the bottom VO_2_ under different phases. The key point is that, on one hand, the thicker VO_2_ layer at the bottom does not interfere with the top layer’s reflection modulation due to the high visible absorption, while on the other hand, the infrared-transparent HfO_2_ ensures that the emissivity tunability of the bottom layer is not affected by the top structure, thus achieving functionally independent control of visible and mid-infrared bands. Figure 1c demonstrates the ideal spectrum for multispectral dynamic camouflage. The device should enable reflectance adjustment in visible band for color camouflage in various environments, while also offering negative emissivity tunability in mid-infrared band that responds to temperature changes for thermal camouflage.

### Performance in dynamic color camouflage

**Fig. 2:**
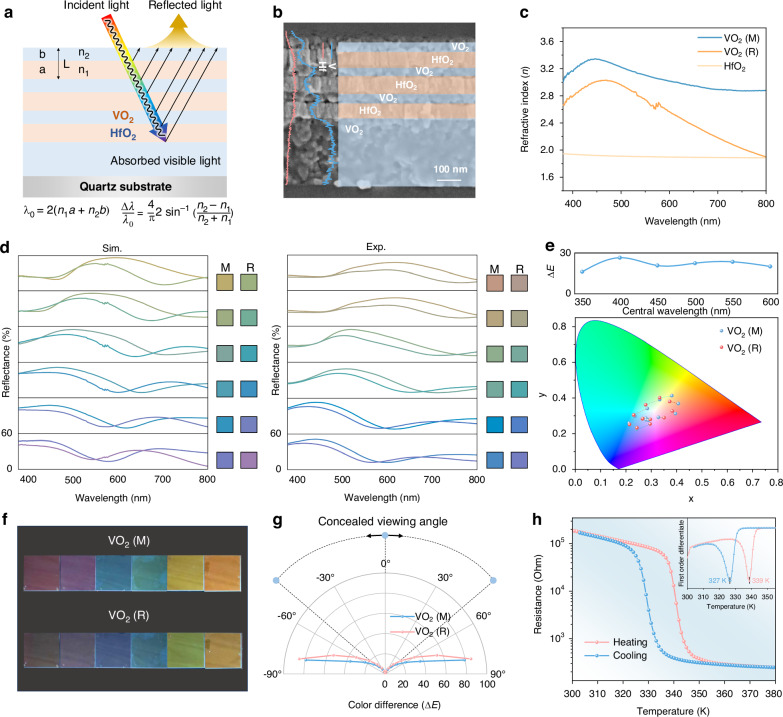
Experimental demonstration of the dynamic color camouflage performance. **a** Designed structure of thermochromic Bragg reflector composed of three periodic layers of VO_2_ and HfO_2_ with corresponding design principle. **b** Cross-section SEM image of MSDR and corresponding element line scan. Herein, the thicknesses of VO_2_ and HfO_2_ in the Bragg reflector are 40 and 65 nm, respectively. And the thickness of bottom VO_2_ is 297 nm. **c** The refractive index (*n*) of HfO_2_, insulating VO_2_ (M) and metallic VO_2_ (R) in the visible band. **d** Simulated (Sim.) and experimental (Exp.) results of the visible spectral reflectance and corresponding reflective colors for Bragg reflector with various thickness combinations of the VO_2_ and HfO_2_ (More details about film thickness combinations are listed in Supplementary Table 4) before and after the phase transition, the central wavelength increases from bottom to top. **e** The reflective colors in CIE 1931 color diagram with various thickness combinations of the VO_2_ and HfO_2_ before and after the phase transition. Upper part: The color difference of the device with different central wavelengths under different states. **f** Photographs of MSDR with different central wavelengths and states. **g** The color difference of MSDR under different states and viewing angles, measured at central wavelength of 600 nm. **h** Resistance hysteresis loop of MSDR and corresponding first-order differentiate curve (inset), which reveal the hysteresis behavior of the device with critical temperature of 339 K and 327 K for heating and cooling process, respectively

The top thermochromic Bragg reflector is composed of three periodic layers of VO_2_ and HfO_2_ (Fig. 2a, b), achieving efficient reflection at central wavelength range. A Bragg reflector typically consists of multiple alternating layers with different refractive indices, where the variations in layer thickness and refractive index induce reflection and interference of light waves between the layers. The central wavelength ${\lambda }_{0}$ and corresponding relative bandwidth ${\Delta \lambda /\lambda }_{0}$ of reflectance spectrum can be calculated as^50,51^:

1 $${\lambda }_{0}=2({n}_{1}a+{n}_{2}b)$$

2 $$\frac{\Delta \lambda }{{\lambda }_{0}}=\frac{4}{\pi }2\,{\sin }^{-1}\left(\frac{{n}_{2}-{n}_{1}}{{n}_{2}+{n}_{1}}\right)$$

Where ${n}_{1}$, ${n}_{2}$ are the refractive index of low-index and high-index materials respectively, $a$ and $b$ are the corresponding thickness. According to the equations, the refractive index of materials will significantly influence the optical performance of the Bragg reflector. As shown in Fig. 2c, VO_2_ exhibits a high refractive index greater than 2.8 in its insulating state, while HfO_2_ demonstrates a low refractive index of less than 1.8. Consequently, the significant refractive index contrast will further enhance the device’s reflectance efficiency. When VO_2_ transitions to its metallic state, the refractive index in visible band exhibits a notable drop, resulting in a blueshift of the central wavelength and relative bandwidth change. Therefore, the reflective color can be dynamically switched with the VO_2_ phase transition happening to adapt to various scenarios.

The central wavelength can be customized by changing the thickness of each film layer. We first listed the layer thickness by varying the central wavelength between 350 nm and 800 nm (More details about film thickness combinations are listed in Supplementary Table 4). Based on the transfer matrix method (TMM) as introduced in Supplementary Note 1 and Supplementary Fig. 1, the reflectance-wavelength curves for different VO_2_/HfO_2_ periods were calculated. Supplementary Figure 2 shows that the optical reflection performance of Bragg reflector within the target wavelength range is significantly enhanced as the number of stacking periods increases (detailed code for calculation is given in Supplementary Note 2). Considering the balance between optical performance and the complexity of subsequent fabrication processes, we ultimately selected the VO_2_/HfO_2_ structure with three period.

To further explore the relationship between film thickness and reflective coloration, the structural colors were simulated across a range of film thicknesses and central wavelengths, spanning from 350 nm to 800 nm (more details about the color calculation are shown in Supplementary Note 3). Figure 2d presents the calculated reflectance spectra at several central wavelengths along with their corresponding reflective colors, demonstrating that a significant hue difference can be achieved not only through the metal-insulator transition but also through combinations of film thickness. Based on this, we further prepared devices with the same thickness as those described above through magnetron sputtering method (More details about the device preparation are described in “Materials and methods”). The measured spectra and reflective colors also align well with the simulation results. The corresponding coordinates are plotted in the CIE 1931 XYZ color space (Fig. 2e), showing a significant color difference (Δ*E*) and an expanded color gamut area covering 57.1% of the standard CMY color space. The photographs shown in Fig. 2f also clearly highlight the diverse colors of the MSDR. Furthermore, the angular dependences of coloration of the proposed camouflage device are further investigated. In camouflage applications, smaller color differences across different viewing angles indicate higher consistency in the target’s appearance, which helps reduce detectability and enhance concealment performance. Figure 2g demonstrates that a wide concealed viewing angle of 100° can be achieved when the color difference remains below 20 when the central wavelength is set as 600 nm, which is attributed to relatively low refractive index contrast between VO_2_ (*n* = 3.02 at 600 nm) and HfO_2_ (*n* = 1.90 at 600 nm). The detailed color modulation of MSDR with different viewing angles for the CIE 1931 color diagram is shown in Supplementary Fig. 3, confirming that the device exhibits color consistency under wide viewing angles. As a comparison, we further calculate the color difference across different viewing angles after substituting the original HfO_2_ with SiO_2_ (*n* = 1.46 at 600 nm), the device shows a larger color difference at the same angles. (More spectral results can be found in Supplementary Fig. 4). When the refractive index difference is smaller, the optical path difference in the multi-layer structure is reduced, and the refraction and reflection of the light wave during its propagation between different materials change more gradually. As a result, the variation in the reflected wavelength with respect to different incident angles is minimized, thus reducing the color difference.

The phase transition temperature of MSDR was determined by measuring the resistance curve of the VO_2_ film during heating and cooling between 300 K and 380 K (Fig. 2h). The corresponding first order differentiate curve indicates that the critical temperature (*T*_c_) of VO_2_ during heating process and cooling process is 327 K and 339 K, respectively (more description about phase transition temperature measure principle can be found in Supplementary Note 4). The phase transition temperature of the thermochromic layer aligns well with that of pure VO_2_^52^, indicating high film quality and purity, more characterization of MSDR is shown in Supplementary Figs. 5–7, which shows the high crystallization and low surface roughness through magnetron sputtering method and post-annealing process. Furthermore, some critical parameters such as transition temperature (*T*_c_), transition width (PTW) and transition rate (PTR) can be further tailored by elements doping^53,54^, enabling temperature-responsive adaptation to meet various requirements.

### Performance in dynamic thermal camouflage

**Fig. 3:**
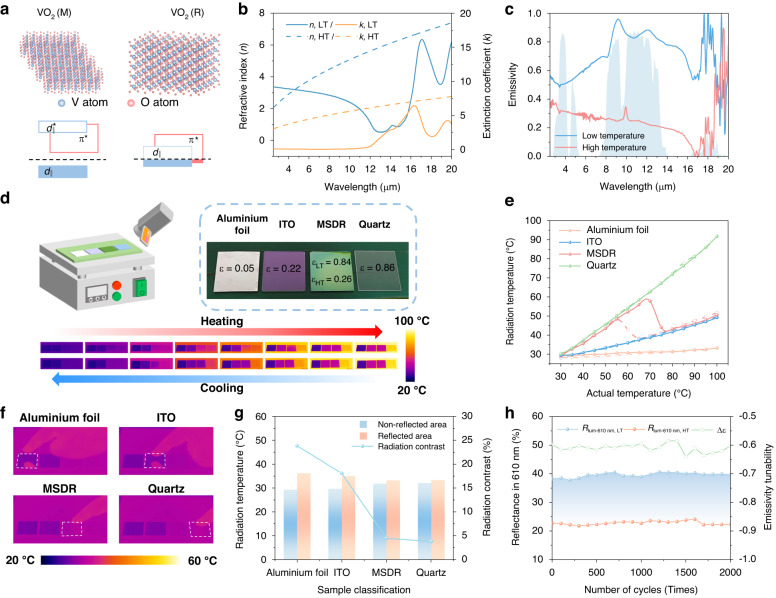
Experimental demonstration of the dynamic thermal camouflage performance. **a** Crystal structures and band structures of VO_2_: the monoclinic insulating phase (M) at low temperature and rutile metallic phase (R) at high temperature. The crystal structures are drawn by VESTS (Ver. 3.90.3a)^58^. **b** The refractive index (*n*) and extinction coefficient (*k*) of insulating VO_2_ (M) and metallic VO_2_ (R) in the mid-infrared band. **c** Thermal radiation spectral emissivity of VO_2_/quartz at low temperature and high temperature. **d** Infrared images of samples at different temperatures from 30 to 100 °C. The samples from left to right are Aluminium foil (*ε* = 0.05), ITO (*ε* = 0.22), MSDR in this work (*ε*_LT_ = 0.84, *ε*_HT_ = 0.26) and quartz (*ε* = 0.86), respectively. The green tape (*ε* = 0.92) is set as background for comparison. **e** The variation curve of radiation temperature of samples with actual temperature. **f** Infrared images of the samples with finger placed above. The thermal radiation of finger can be clearly reflected on low-emissivity samples (Aluminium foil, ITO) compared with high-emissivity samples (MSDR, quartz) under room temperature conditions. **g** The radiation temperature and corresponding radiation contrast between non-reflected and reflected areas of the samples with a finger placed above. The calculation of radiation contrast: (*T*_reflected_ − *T*_non-reflected_)/*T*_non-reflected_ * 100%, where *T*_reflected_ and *T*_non-reflected_ represent the radiation temperature of the finger-reflected area and surface area without finger-reflected respectively. **h** The results of reflectance in 610 nm and emissivity tunability after 2000 heating and cooling cycles

As for the mid-infrared band, VO_2_ also shows significant changes in optical properties during its phase transition (Fig. 3a). VO_2_ exhibits high infrared transparency in the insulating state (*n* > *k*, *k* ≈ 0), while it becomes more reflective in its metallic state (*n* < *k*) at high temperatures (Fig. 3b). In the insulating phase, the bandgap of VO_2_ is approximately 0.6–0.7 eV^35,53^. Since most electrons are confined to the valence band with nearly no free electrons in the conduction band, it exhibits low infrared reflectivity. After MIT, the valence band overlaps with the conduction band, eliminating the bandgap entirely. Electrons in the valence band become free to move, forming a high-density free electron gas characteristic of the metallic state. As a result, less infrared energy is absorbed due to the larger plasma energy of free electrons (≈1.2 eV) significantly exceeds that of thermal infrared photons (≈0.1 eV)^13,55^. The larger *k* in the metallic state compared to the insulating state also highlights the significant change of infrared transparency and reflectance.

With its ability to regulate the mid-infrared band, VO_2_ can be combined with high-emissivity materials to achieve a device with negative emissivity tunability (Δ*ε* = *ε*_HT_ − *ε*_LT_ < 0), exhibiting high emissivity at low temperatures and low emissivity at high temperatures, which is the key property for temperature-adaptive thermal camouflage. As shown in Supplementary Fig. 8, the maximum electric-field amplitude occurs when VO_2_ is in its insulating state and transparent in the mid-infrared band. Under this condition, the structure exhibits a high electric-field amplitude, indicating high emissivity at low temperature. When VO_2_ turns into metallic state, the electric field amplitude is substantially reduced as a result of the strong infrared reflectivity of VO_2_ and most incident electromagnetic wave is reflected, thus the device shows a low emissivity state. Herein, we selected quartz as the bottom high-emissivity material, which not only offers high emissivity (*ε* = 0.86) but also functions as the substrate. After optimizing film thickness of VO_2_/quartz structure (Supplementary Fig. 9), a significant emissivity tunability of −0.6 can be achieved in the 8–14 μm (Fig. 3c). Most importantly, MSDR exhibits similarly significant emissivity tunability (Δ*ε*_8–14 μm_ = −0.58) because the IR-transparent Bragg reflector has negligible impact on the bottom dynamic infrared camouflage layer (Supplementary Fig. 10). The underlying mechanism is primarily attributed to the pronounced change in skin depth of the VO_2_-based device between its low and high temperature states (more descriptions of skin depth analysis are shown in Supplementary Note 5), resulting in electromagnetic waves being either absorbed by the high-emissivity substrate or reflected by the highly reflective VO_2_ layer, depending on the device state.

To comprehensively evaluate the thermal camouflage performance of the MSDR, we selected low emissivity films Aluminium foil (*ε* = 0.05) and transparent ITO (*ε* = 0.22) as the static thermal camouflage devices for comparison. And we chose quartz (*ε* = 0.86) as reference sample and tape (*ε* = 0.92) as background. We further took infrared images through infrared camera at temperatures ranging from 30 °C to 100 °C, and corresponding radiation temperatures are shown in Fig. 3d. More detailed results can be found in Supplementary Fig. 11 and Fig. 3e, it can be seen that at the low temperature, MSDR shows the same radiation temperature with quartz, matching the background. With the temperature increases, the radiation temperature gradually decreases due to the negative emissivity tunability (Δ*ε* < 0), and MSDR presents the similar radiation temperatures with low-emissivity ITO at the high temperature. When the target temperature is close to the ambient temperature, the high emissivity of MSDR matches the ambient emissivity, resulting in similar radiative temperatures for both. As the target temperature increases, MSDR transitions to a low-emissivity state, suppressing the rise in radiation temperature and minimizing the radiation temperature difference with ambient, thereby preventing infrared detection. When the actual temperature of object reaches 100 °C, the radiative temperature in the area covered by MSDR is approximately 50 °C, exhibiting a significant halving effect. As a result, the target can seamlessly blend into the background under infrared detection, regardless of whether it is at a low or high temperature.

Figure 3e shows the excellent camouflage effect presented by Aluminium foil in a hot temperature, but we aim to determine whether maintaining consistently low emissivity is suitable for infrared camouflage across various scenarios. The primary challenge lies in the susceptibility of low-emissivity devices to interference from external heat sources, which can compromise their camouflage performance^17^. To validate this, we positioned a finger at an oblique angle above all samples at room temperature (Fig. 3f). Although all samples could blend with the background thermal radiation at room temperature, under the influence of a finger with higher radiation temperature, low-emissivity (high infrared-reflective) devices such as Aluminium foil and ITO strongly reflected the finger’s radiative energy, thereby mirroring its shape. In contrast, high-emissivity samples such as MSDR and quartz can significantly mitigate this adverse effect. Figure 3g shows that the area radiation contrast of MSDR is much lower than low-emissivity Aluminium foil and ITO. If the ambient temperature is lower (e.g., winter, night or snowy ground), the presence of external heat sources (e.g., human, vehicle or flame) may cause hot spots on low emissivity surface to disrupt the camouflage performance. Therefore, compared with static camouflage technology, MSDR will not only enhance dynamic camouflage performance across diverse environments, but also mitigates interference from additional heat sources.

To further investigate the long-term performance for practical use, MSDR was heated under an infrared lamp and cooled in the natural environment (more details for experiment setup can be found in Supplementary Fig. 12). After measuring the reflectance in 610 nm and thermal radiation spectral emissivity tunability after 2000 heating and cooling cycles. No obvious deterioration of optical properties can be found in Fig. 3h. Minor fluctuations may be due to unavoidable variations in the testing environment. Thanks to the excellent films quality and the robust switching durability of VO_2_^56^, the device demonstrates reliable performance in complex and dynamic real-world environments.

### Device improvements for advanced camouflage

**Fig. 4:**
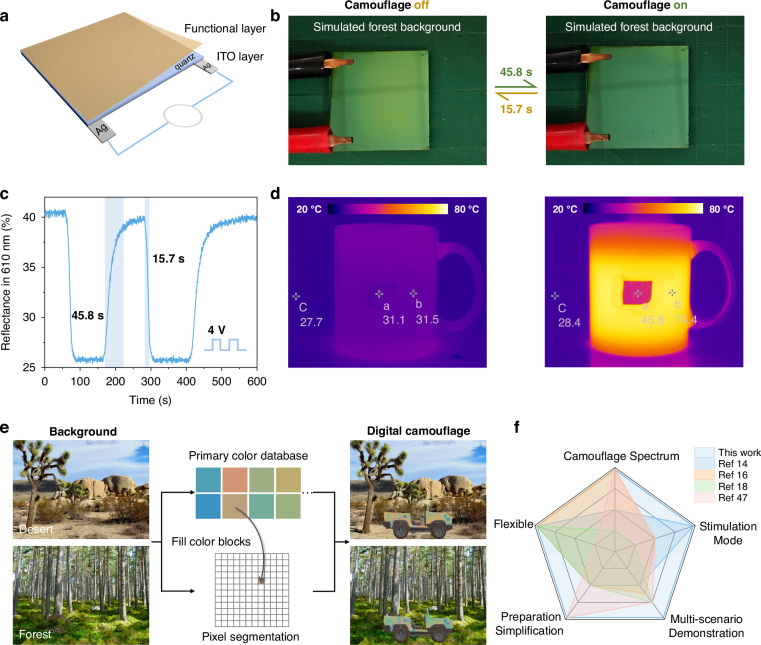
Experimental demonstration of device improvements for advanced camouflage. **a** Schematic diagram for an active-response device controlled by a voltage input. ITO layer was deposited on the backside of device and connected to Ag electrodes for voltage input. **b** Photographs of MSDR camouflage in the dark-green simulated forest background with the voltage on and off. **c** The measured camouflage on and off times for device controlled by a voltage input through the measurement of the reflectance in 610 nm. The shaded area indicates the time to reach 90% of maximum or minimum reflectance. **d** Infrared images before and after pouring hot water into the cup covered with flexible MSDR. “a”, “b”, “c” letters represent the radiation temperatures of MSDR-covered area, without MSDR-covered area and background respectively. **e** Conceptual demonstration of digital camouflage based on MSDR. The desert and forest photo are downloaded from website (https://www.pexels.com/) with permission under a Creative Commons license CC0. The final digital camouflage patterns can be gotten through Neighboring Color Block Camouflage Algorithm. **f** The radar chart displays a comparative analysis of various key factors related to MSDR and current dynamic camouflage technologies. MSDR showcases superior properties in all respects in the comparison

For practical multispectral camouflage applications, the device requires further optimization to fully demonstrate its potential for real-world deployment. The first challenge we face is that VO_2_ exhibits a fixed phase transition temperature. Although elements doping can tailor the phase transition temperature, it still lacks flexibility in complex and variable environments. In addition to leveraging the temperature-adaptive properties of VO_2_ for passive response, employing electric fields to actively regulate its phase transition process is equally significant. Herein, we deposited conductive ITO film on the backside of device without influencing the camouflage performance as shown in Fig. 4a. Thus, the MIT process can be actively controlled through Joule heating by voltage input. Figure 4b, c illustrate that by inputting 4 V voltage, the switching times for device’s “camouflage on” and “camouflage off” states are relatively fast, measuring 45.8 s and 15.7 s, respectively. The photographs also demonstrate the device’s color transition from an earthy yellow to a forest green, enabling it to blend into the simulated forest background.

What’s more, the flexibility of device is another key factor for practical application. Flexible camouflage devices are highly valued for their ability to conform to complex surfaces and adapt to dynamic targets. Their flexibility ensures a close fit to irregular structures, minimizing gaps that could expose infrared signals, while maintaining stable camouflage performance during movement, making them versatile for various applications. Limited by the rigid quartz, the device we prepared lacks flexibility. Herein, we chose another high-emissivity and flexible substrate polyimide (PI) to replace the rigid quartz through process optimization, and the corresponding spectra (more details about the measured visible spectral reflectance and emissivity are shown in Supplementary Figs. 13 and 14) also shows that the flexible device achieves significant color change and excellent Δ*ε*_8–14 μm_ of −0.48. Furthermore, we affixed the flexible device to a ceramic cup and took infrared images before and after filling the cup with hot water (Fig. 4d and Supplementary Fig. 15). The images demonstrate that the flexible device can match the radiative power of the cup at low temperatures, while effectively shielding the high radiative power generated by the cup at high temperatures. To comprehensively evaluate the durability performance of the flexible device, we measured its optical properties after different bending 20,000 cycles, the mechanically folded flexible device shows consistent performance without obvious optical degradation (more description and results about the folding tests are shown in Supplementary Note 6 and Supplementary Figs. 16 and 17). Besides, the flexible device with the sizes of 25 × 25 mm and 55 × 55 mm shows excellent outstanding in multi-sized fabrication (Supplementary Fig. 18), which is not only capable of covering various targets but also provides excellent camouflage performance. Although slight surface unevenness is observed in the larger-sized samples (Supplementary Fig. 19), this is attributed to the thermal expansion of the substrate during the post-annealing process, which increases the likelihood of warping. This issue can be mitigated by optimizing the substrate material preparation to achieve both good thermal resistance and a certain degree of flexibility, as well as by improving the VO_2_ deposition process to allow for lower heat treatment temperatures^56^. All the strategies can be applied to balance the optical performance of the film and the uniformity of the device, which opens up broader possibilities for the development of large-scale flexible devices for camouflage applications.

Besides, based on current performance of MSDR, the potential power input for Joule heating is concerned. By controlling the input voltage switch strategy, the VO_2_-based device can be actively modulated. As shown in Supplementary Fig. 20, a voltage of 2.5 V has reached the phase transition temperature, and higher heating speed can be reached by improving voltage input. Based on the sample size (50 × 30 mm) and measured power consumption of 0.56 W, the corresponding power density is calculated to be 373.3 W m^−2^. The additional energy input at this level is generally manageable for the covered object. And these advantages may offset the power requirement to some extent, especially in scenarios where real-time thermal modulation is prioritized. Moreover, while Joule heating provides an effective means of active modulation, the device also exhibits passive responsiveness, which can partially mitigate the energy consumption during operation. Most importantly, higher heating efficiency can be achieved by replacing the ITO with lower resistivity film, such as Pt, Au or Ti, which can achieve the same level of thermal power with reduced electrical driving requirements.

Digital camouflage is a pattern composed of pixelated square color blocks, arranged in a random sequence to break up the target’s outline and enhance concealment and camouflage effectiveness. The design of digital camouflage can be adjusted based on different operational environments, with colors and pattern details optimized accordingly, allowing it to achieve effective concealment under various conditions. As previously discussed, MSDR can not only change colors under different states but also customize a range of color combinations by varying the material thickness. On one hand, MSDR can customize a range of color combinations by varying the material thickness, which make sure that camouflage effect can be achieved through the combination of color blocks in a fixed scenario. To some extent, it achieves the same effect as current static camouflage, which is essential for camouflage for the specific scenario. On the other hand, MSDR can dynamically change reflective colors under different states, making the target to be concealed in different scenarios. Therefore, we can design a digital camouflage system by using the camouflage devices, where the combination of different initial colors forms block patterns, and the color switching enables adaptation to different scenarios, thereby enhancing camouflage reliability. Figure 4e provides a conceptual demonstration of the digital camouflage. A desert and forest are selected as the backgrounds, the color information of the images is stored and divided into multiple pixel blocks. Several primary color combinations are selected according to our previous research shown in Fig. 2d, Neighboring Color Block Camouflage Algorithm is applied to fill the primary colors into the corresponding pixel blocks and match them with the original colors of the images (the workflow of Neighboring Color Block Camouflage Algorithm is presented in Supplementary Fig. 21). The final output digital camouflage images can not only be applied to camouflage in specific scenes but also exhibit adaption to diverse environments, demonstrating the significant potential of MSDR in advanced camouflage technology.

Furthermore, to illustrate the advantages highlighted in MSDR, various factors including camouflage spectrum, stimulation mode, flexible, preparation simplification and multi-scenario demonstration are applied for the comparison with current dynamic camouflage technology (Fig. 4f). For detailed summary and evaluation criteria, see Supplementary Note 7 and Supplementary Table 5. Our research demonstrates significant advancements over current dynamic camouflage technologies.

## Discussion

In summary, we present a multispectral dynamic regulator for color-thermal camouflage by applying thermochromic VO_2_ and visible-infrared transparent HfO_2_. Based on the design of Bragg reflector and negative emissivity tunability layer, MSDR shows a wide color gamut while functional independently achieving highest negative emissivity tunability of −0.58 so far, marking the first experimental realization of multispectral camouflage across both visible and infrared spectra among the VO_2_-based emissivity tunability devices. Moreover, by improving the long-cycle stability device to achieve active-passive dual-mode response and flexibility, we further evaluate the camouflage performance of multispectral dynamic regulator in complex real-world environments. Through the demonstration of the digital camouflage concept, we highlighted the immense potential of MSDR for practical applications.

With the advancement of multispectral detection technology and the increasing complexity of camouflage scenario requirements, the demand for multispectral dynamic camouflage technology is growing increasingly urgent. The integration of advanced camouflage technologies with materials science, optoelectronic detection, computational algorithms, and physics will further accelerate interdisciplinary interaction and collaborative development. We expect that the designed device can not only advance the progress of multispectral dynamic camouflage, but also expand to information anti-counterfeiting, flexible electronics and thermal management.

## Materials and methods

### VO_2_ layer deposition

The quartz substrates were washed in ethanol, acetone and deionized water, then blown and dried with a high-pressure nitrogen gun. All the layers were deposited with the physical vapor deposition (PVD) method of magnetron sputtering. The VO_2_ films were deposited by direct current (DC) magnetron sputtering using a 4-in. diameter V_2_O_3_ target (99.9% purity) in a 9 mTorr argon and oxygen mixed atmosphere (Ar/O_2_ = 48:2) at room temperature with the sputtering power of 200 W, with the deposition rate of 5.9 nm min^−1^. After sputtering VO_2_ layer, the samples were post-annealed in a chamber at 450 °C with the vacuum of 5 Torr for 5 min, followed by natural cooling.

For the flexible device fabrication, polyimide (PI) was selected as substrate. The key parameters of the sputtering process are consistent with those on the quartz substrates and the main difference lies in the post-annealing process. The samples grown on PI were post-annealed in a chamber at 400 °C with the vacuum of 5 Torr for 5 min, followed by natural cooling.

### HfO_2_ layer deposition

The HfO_2_ layers were prepared by radio frequency (RF) magnetron sputtering using a 4-in. diameter HfO_2_ target (99.99% purity) in a 7 mTorr pure argon atmosphere at room temperature with the sputtering power of 200 W, with the deposition rate of 4.2 nm min^−1^.

### Sample characterization

The crystalline phases of films were characterized by X-ray diffraction (XRD, BRUKER AXS GMBH D8 ADVANCE). The morphology of the films and element line scan were measured by scanning electron microscopy (SEM, FEI Verios G4) and atomic force microscopy (AFM, NT-MDT NTEGRA). The transmittance spectra (350–2600 nm) were measured by using a UV–Vis–NIR spectrophotometer (Hitachi U-4100) coupled with an integrating sphere. The infrared spectra (2.5–20 μm) were measured by using a Fourier transform infrared spectrometer (FTIR, Thermo Scientific NICOLET Is10) coupled with a diffuse gold surface. The IR images were taken by infrared camera (InfRec R300SR) with the spectral range of 8–14 μm, to minimize the reflection signals from the surroundings, the viewing angle was fixed as 30°. The thermal hysteresis loops were recorded by measuring the resistance of the samples on the heating stage located in vacuum cryostats (VPF-100) with the temperature range from 300 K to 380 K.

### Emissivity calculation

According to Kirchhoff’s law, the emissivity (*ε*_8–14 μm_) and emissivity tunability (Δ*ε*_8–14 μm_) in the range of 8–14 μm can be calculated by the reflectance spectra using the following equations:

3 $$B(\lambda ,T)=\frac{2h{c}^{2}}{{\lambda }^{5}}\frac{1}{{e}^{\frac{{hc}}{\lambda {kT}}}-1}$$

4 $${\varepsilon }_{8{{\mbox{-}}}14{\rm{\mu }}{\rm{m}}}=\frac{{\int }_{8\mu m}^{14\mu m}(1-R(\lambda ))B(\lambda ,T)d(\lambda )}{{\int }_{8\mu m}^{14\mu m}B(\lambda ,T)d(\lambda )}$$

5 $${\varDelta \varepsilon }_{8{{\mbox{-}}}14{\rm{\mu }}{\rm{m}}}={\varepsilon }_{{ht}}-{\varepsilon }_{{lt}}$$

where *B*(*λ*, *T*) represents the thermal radiation power density of the blackbody for a temperature *T*, *h* is the Planck constant, *c* represents the speed of light, *k* is the universal Boltzmann constant, and *R*(*λ*) is the reflectance at the wavelength *λ*. lt and ht represent 30 and 100 °C, respectively.

### Optical simulations

The results of reflectance-wavelength curves for different VO_2_/HfO_2_ periods shown in Supplementary Fig. 2 was calculated by transfer matrix method (TMM), which can be found detailed description in Supplementary Notes 1 and 2. The visible reflectance and emissivity results are performed by Essential Macleod. The corresponding optical constant are collected from the ref.^57^, website (https://refractiveindex.info/) and our experimental measurement.

### Numerical simulations

The multi-layer structure is built according to the fabricated thickness. As for the boundary conditions, periodic boundary conditions are applied in both the x- and y-directions, and perfectly matched layer are applied in the z-direction, the mesh accuracy is set to 8. A plane wave under normal incidence is used as the source, while a frequency domain profile monitor is configured to capture the electric field distribution along the *z*-axis of the structure. The refractive index of VO_2_, HfO_2_ are consistent with the above introduction.

## Supplementary information

Supplementary Information

## Data Availability

The authors declare that all data supporting the findings of this study can be found within the paper and its Supplementary information file. Additional data supporting the findings of this study are available from the corresponding author upon reasonable request.
