# Supplementary material for: Color-thermal multispectral camouflage with VO2-based dynamic regulator
Source: Light Sci Appl. 2025 Sep 10;14:313. doi: 10.1038/s41377-025-01968-x (PMC12423319; doi:10.1038/s41377-025-01968-x)
Supplement: Supplementary file 1 — Supplementary Information [file 41377_2025_1968_MOESM1_ESM.docx]

Supplemental Information for

**Color-Thermal Multispectral Camouflage with VO_2_-based Dynamic Regulator**

Chengcong Li^1,2^, Cuicui Cao^1,2^, Zhongshao Li^1,2^, Zewei Shao^1,2^, Fei Cao^1,3^, Genshui Wang^1,3^, Ping Jin^1^, Hongjie Luo^4^ and Xun Cao^1,2,*^

^1^State Key Laboratory of High Performance Ceramics and Superfine Microstructure, Shanghai Institute of Ceramics, Chinese Academy of Sciences, Shanghai 200050, China.

^2^Center of Materials Science and Optoelectronics Engineering, University of Chinese Academy of Sciences, Beijing 100049, China.

^3^The Key Lab of Inorganic Functional Materials and Devices, Shanghai Institute of Ceramics, Chinese Academy of Sciences, Shanghai 200050, China.

^4^Institute for the Conservation of Cultural Heritage, Shanghai University, Shanghai 200444, China.

*Corresponding Authors: <cxun@mail.sic.ac.cn> (X.C.)

**Content**

Supplementary Notes 1–7

Supplementary Figures 1–21

Supplementary Tables 1–5

Supplementary References

**Supplementary Note 1: The Transfer Matrix Method for reflectance calculation**

The Transfer Matrix Method (TMM) is a widely used approach for simulating the optical properties of thin films. As a special type of periodically arranged thin-film structure (**Fig. S1**), the algorithm based on Bragg reflector can be simplified^1^.

The relationship of refractive index and position can be described:

| $n(x)=\left\{ \begin{aligned} n_{2}, 0<x<b \\ &n_{1}, b<x<L \end{aligned} \right.$ | (1) |
| --- | --- |
| $n(x+L)=n(x)$ | (2) |

where *n*_1_, *n*_2_ are the refractive index of low-index and high-index materials respectively, *a* and *b* are the corresponding thickness, *L* is the length of the single periodic structure. The matrix elements can be calculated from:

| $A=e^{-ik_{1x}a}[\cos k_{2x}b-\frac{1}{2}i(\frac{n_{2}^{2}}{n_{1}^{2}}\frac{k_{1x}}{k_{2x}}+\frac{n_{1}^{2}}{n_{2}^{2}}\frac{k_{2x}}{k_{1x}})\sin k_{2x}b]$ | (3) |
| --- | --- |
| $B=e^{ik_{1x}a}[-\frac{1}{2}i(\frac{n_{2}^{2}}{n_{1}^{2}}\frac{k_{1x}}{k_{2x}}-\frac{n_{1}^{2}}{n_{2}^{2}}\frac{k_{2x}}{k_{1x}})\sin k_{2x}b]$ | (4) |
| $C=e^{-ik_{1x}a}[\frac{1}{2}i(\frac{n_{2}^{2}}{n_{1}^{2}}\frac{k_{1x}}{k_{2x}}-\frac{n_{1}^{2}}{n_{2}^{2}}\frac{k_{2x}}{k_{1x}})\sin k_{2x}b]$ | (5) |
| $D=e^{ik_{1x}a}[\cos k_{2x}b+\frac{1}{2}i(\frac{n_{2}^{2}}{n_{1}^{2}}\frac{k_{1x}}{k_{2x}}+\frac{n_{1}^{2}}{n_{2}^{2}}\frac{k_{2x}}{k_{1x}})\sin k_{2x}b]$ | (6) |

where wave vector can be given by:

| $k_{\alpha x}=(\omega/c)n_{\alpha}$, $\alpha=1, 2$ | (7) |
| --- | --- |

The coefficient of reflection is simply

| $r_{N}={(b_{0}/r_{N})}_{b_{N}=0}$ | (8) |
| --- | --- |

The column vectors of the equivalent layers are given as:

| $\binom{a_{0}}{b_{0}}=\left( \begin{matrix} A & B \\ C & D \end{matrix} \right)^{N}\binom{a_{N}}{b_{N}}$ | (9) |
| --- | --- |

The *N*th power of an unimodular matrix can be given by:

| $\left( \begin{matrix} A & B \\ C & D \end{matrix} \right)^{N}=\left( \begin{matrix} AU_{N-1}-U_{N-2} & BU_{N-1} \\ CU_{N-1} & DU_{N-1}-U_{N-2} \end{matrix} \right)$ | (10) |
| --- | --- |

Where

| $U_{N}=\sin\left( N+1 \right)KL/\sin KL$ | (11) |
| --- | --- |
| $K={(1}/L)\cos^{-1} [\frac{1}{2}(A+D)]$ | (12) |

The coefficient of reflection is

| $r_{N}=CU_{N-1}/(AU_{N-1}-U_{N-2})$ | (13) |
| --- | --- |

Thus, the reflectivity is obtained by taking the absolute square of *r*_N_,

| $\left\vert r_{N} \right\vert^{2}=\frac{\left\vert C \right\vert^{2}}{\left\vert C \right\vert^{2}+\left( \sin KL/\sin NKL \right)^{2}}$ | (14) |
| --- | --- |

**Supplementary Note 2: The detailed code for periods calculation**

To decide an optimal period number with the consideration of both optical performance and the complexity of subsequent fabrication processes, we set the central wavelength to 500 nm and varied the number of periods from 2 to 5. The MATLAB code based on the Transfer Matrix Method as introduced in **Supplementary Note 1** is presented below.

clear;clc

for N=2:5; *%N is the period numbers from 2 to 5.*

n_L=1.9; *%* *n_L is the refractive index of HfO_2_ in 500 nm.*

n_H=3.22; *%* *n_H is the refractive index of VO_2_ in 500 nm.*

a_L=65.46; *% n_L is the calculated thickness of HfO_2_.*

a_H=38.81; *% n_H is the calculated thickness of VO_2_.*

L=a_L+a_H; *% L is the periodic thickness of Bragg reflector.*

lambda=300:10:850; *% lambda is the incident wavelength.*

k_L=2*pi*n_L./lambda;

k_H=2*pi*n_H./lambda;

A=exp(-1i*a_L*k_L).*(cos(k_H*a_H)-(1i/2)*((n_H^2.*k_L)./(n_L^2.*k_H)+(n_L^2.*k_H)./(n_H^2.*k_L)).*sin(k_H*a_H));

B=exp(1i*a_L*k_L).*(-(1i/2)*((n_H^2.*k_L)./(n_L^2.*k_H)-(n_L^2.*k_H)./(n_H^2.*k_L)).*sin(k_H*a_H));

C=exp(-1i*a_L*k_L).*((1i/2)*((n_H^2.*k_L)./(n_L^2.*k_H)-(n_L^2.*k_H)./(n_H^2.*k_L)).*sin(k_H*a_H));

D=exp(1i*a_L*k_L).*(cos(k_H*a_H)+(1i/2)*((n_H^2.*k_L)./(n_L^2.*k_H)+(n_L^2.*k_H)./(n_H^2.*k_L)).*sin(k_H*a_H)); *%A, B, C, D represent the matrix elements.*

K=(1/L)*acos((A+D)/2);

R=abs(C).^2./(abs(C).^2+(sin(K*L)./sin(N*K*L)).^2);; figure (1) *% R is the reflectivity.*

R=real(R');

plot(lambda,R,'LineWidth',1); hold on *% Input final results.*

end;

The calculated reflectivity can be found in **Fig. S2**.

**Supplementary Note 3: Principle of color calculation**

In the CIE 1931 XYZ color space, the stimulus quantities of the three primary colors are expressed as tristimulus values *X*, *Y*, and *Z*, which can be given by^2^:

| $X=\sigma\int_{380nm}^{780nm} D_{65}\left( \lambda\right)R\left( \lambda\right)\bar{x}\left( \lambda\right)d\lambda$ | (15) |
| --- | --- |
| $Y=\sigma\int_{380nm}^{780nm} D_{65}\left( \lambda\right)R\left( \lambda\right)\bar{y}\left( \lambda\right)d\lambda$ | (16) |
| $Z=\sigma\int_{380nm}^{780nm} D_{65}\left( \lambda\right)R\left( \lambda\right)\bar{z}\left( \lambda\right)d\lambda$ | (17) |

where $D_{65}\left( \lambda\right)$ is the spectral power distribution of the standard illuminants *D*65 and $R\left( \lambda\right)$ represents the object reflectance. $\bar{x}\left( \lambda\right)$, $\bar{y}\left( \lambda\right)$ and $\bar{z}\left( \lambda\right)$ are the color matching functions. $\sigma$ is a normalizing factor defined as:

| $\sigma=\frac{100}{\int_{380nm}^{780nm} D_{65}\left( \lambda\right)\bar{y}\left( \lambda\right)d\lambda}$ | (18) |
| --- | --- |

The color chromaticity can be determined by the normalized parameters *x*, *y*, and *z*:

| $x=\frac{X}{X+Y+Z}$ | (19) |
| --- | --- |
| $y=\frac{Y}{X+Y+Z}$ | (20) |
| $z=\frac{Z}{X+Y+Z}$ | (21) |

To calculate the color difference (Δ*E*), CIE 1976 L^*^a^*^b^*^ color space is utilized, where *L*^*^ represents the lightness, *a*^*^ represents redness and greenness, and *b*^*^ represents yellowness and blueness:

| $L^{*}=116f\left( \frac{Y}{Y_{0}} \right)-16$ | (22) |
| --- | --- |
| $a^{*}=500\left[ f\left( \frac{X}{X_{0}} \right)-f\left( \frac{Y}{Y_{0}} \right) \right]$ | (23) |
| $b^{*}=200\left[ f\left( \frac{Y}{Y_{0}} \right)-f\left( \frac{Z}{Z_{0}} \right) \right]$ | (24) |

where $X_{0}$, $Y_{0}$ and $Z_{0}$ represent the tristimulus values of the white object.

| $f\left( t \right)=\left\{ \begin{aligned} t^{1/3}, t>\left( \frac{24}{116} \right)^{3} \\ \frac{841}{108}t+\frac{16}{116},t\leq\left( \frac{24}{116} \right)^{3} \end{aligned} \right.$ | (25) |
| --- | --- |

The color difference can be calculated from:

| $\Delta E=\sqrt{{(\Delta L^{*})}^{2}+{(\Delta a^{*})}^{2}+{(\Delta b^{*})}^{2}}$ | (26) |
| --- | --- |

**Supplementary Note 4: Principle of phase transition temperature measure**

VO_2_ is a representative first-order phase transition material and there are some methods to character its phase transition process. Resistance-temperature measurement is commonly used to analyze the phase transition process. During the heating process, VO_2_ thin films exhibit a characteristic metal-insulator transition (MIT), accompanied by a pronounced change in electrical resistance. At temperatures below the phase transition, VO_2_ remains in the monoclinic insulating phase, characterized by high electrical resistance. As the temperature increases and approaches the critical region, a structural transformation occurs toward the rutile metallic phase, resulting in a sharp drop in resistance by several orders of magnitude within a narrow temperature range. This abrupt change signifies the transition from the insulating to the metallic state. Upon cooling, VO_2_ reverts from the metallic to the insulating phase, with the resistance rapidly increasing, forming a distinct thermal hysteresis loop.

The steep resistance variation within the transition region reflects the underlying phase transition behavior and is typically characterized by the temperature corresponding to the minimum of the first derivative of the resistance-temperature curve, which represents the point of maximum rate of change in electrical properties during the transition. This temperature point is defined as the critical temperature (*T*_c_), which is typically considered the phase transition temperature of VO_2_ during the heating cycle.

**Supplementary Note 5: Skin depth analysis**

In order to analyze whether the multi-layer thickness is suitable for design, the skin depth analysis is performed on the device structure. Skin depth ($\delta$) refers to the distance within a conducting material where an electromagnetic wave can effectively penetrate before its intensity significantly decays, which is defined as:

| $\delta=\sqrt{\frac{2}{\omega\mu\sigma}}=\sqrt{\frac{1}{f\pi\mu\sigma}}$ | (27) |
| --- | --- |

where $\omega$ and $f$ are angular frequency and frequency of the electromagnetic wave, $\mu$ is the permeability of the material (${\mu=\mu}_{r}*\mu_{0}$, $\mu_{r}$ is relative permeability and $\mu_{0}$ is vacuum permeability), $\sigma$ is the electrical conductivity of the material. As the non-magnetic material, the relative permeability of VO_2_ is 1, and the corresponding permeability is 4π×10^-7^ H m^-1^. And the electrical conductivity of insulating and metallic VO_2_ is 1 and 10^5^ S m^-1^ respectively^3^. So the skin depth of the device under the monitoring wavelength of 10 μm can be calculated as 2.9 μm and 0.29 μm for low and high temperatures. HfO_2_ acts as an insulator, enabling deep penetration of electromagnetic waves. Thus, the thickness of the device is well-suited for the design, with a total VO_2_ thickness of 417 nm. Under insulating state, VO_2_ allows the electromagnetic wave to pass through the multi-layer and to be absorbed by the bottom substrates. And in the metallic state, it can also block the electromagnetic wave transmission and reflect the electromagnetic wave.

**Supplementary Note 6: Sample folding tests**

The flexible sample was attached on two metal arms, whose movement can be controlled by computer through software. By providing the movement distance, rate and cycling numbers, the folding tests will be performed automatically. The visible spectral reflectance and thermal radiation spectral emissivity were measured at the cycle number of 1000, 2000, 3000, 4000, 5000, 7500, 10000, 12500, 15000, 17500 and 20000.

More detailed optical properties of the folding tests have been shown in **Fig. S16**.

**Supplementary Note 7: Evaluation criteria for dynamic camouflage technology**

**(1) Camouflage spectrum:** This parameter refers to the spectral range in which the device achieves camouflage. And vis, MIR represent visible and mid-infrared bands respectively. The broader the camouflage spectrum, the more resilient the device becomes in multispectral detection scenarios.

**(2) Stimulation mode:** This metric is used to describe the stimulus types by which the device achieves optical performance switching, such as temperature (thermal) or electric field (electrical). The more diverse the stimulation modes, the more adaptable the device becomes in real-world scenarios.

**(3) Multi-scenario demonstration:** In addition to characterizing the optical performance of the fabricated devices, it is essential to provide scenario demonstrations to visually illustrate their practicality.

**(4) Preparation simplification:** In the device fabrication process, fewer procedural steps (single-step or multi-step fabrication) and easier fabrication technology highlight the simplicity and efficiency of the workflow, typically regarded as an optimized fabrication approach.

**(5) Flexible:** The device’s flexibility allows it to accommodate a wide range of target geometries, which enhances the adaptability and applicability of camouflage technology.

More detailed description of these evaluation criteria has been listed in **Table S4**.

**Fig. S1 Schematic drawing of a Bragg reflector structure.** The structure has *N* layers and the ambient environment is represented by 0. *a* and *b* represent the transmission and reflection intensity respectively.

**Fig. S2 The calculated reflectivity of the Bragg reflector with the different periods (*N*=2, 3, 4, 5).** The higher periods exhibit superior reflectivity and spectral selectivity. But when the periods exceed 3, the difference is not obvious.

**Fig. S3 The color modulation of MSDR with different viewing angles for the CIE 1931 color diagram (the viewing angles from 0 to 80°).**

**Fig. S4 The color difference curve across different viewing angles with the Bragg reflector composed of VO_2_/HfO_2_ and VO_2_/SiO_2_.** Large color difference can be found for the combination of VO_2_ and SiO_2_ compared to that of VO_2_ and HfO_2_.

**Fig. S5 Original SEM image of cross-sectional MSDR and corresponding element line scan.**

**Fig. S6 XRD patterns of VO_2_ on quartz and MSDR device.**

**Fig. S7 3D AFM image for MSDR device and corresponding root-mean-square (RMS) roughness.** The RMS of MSDR is 3.492 nm, indicating the smooth surface of MSDR with a thickness of approximately 600 nm.

**Fig. S8** **The electric field distribution of the VO_2_-based multi-layer structure in low (a) and high (b) temperatures**. The detected wavelength is set as 10 μm.

**Fig. S9 The simulated results of emissivity tunability in 8–14 μm with different thickness of VO_2_.** With the thickness of VO_2_ increases, the emissivity tunability also increases before 300 nm.

**Fig. S10 Thermal radiation spectral emissivity of the multi-layer film** **at low temperature and high temperature.** The emissivity tunability can reach -0.58 in the atmospheric window.


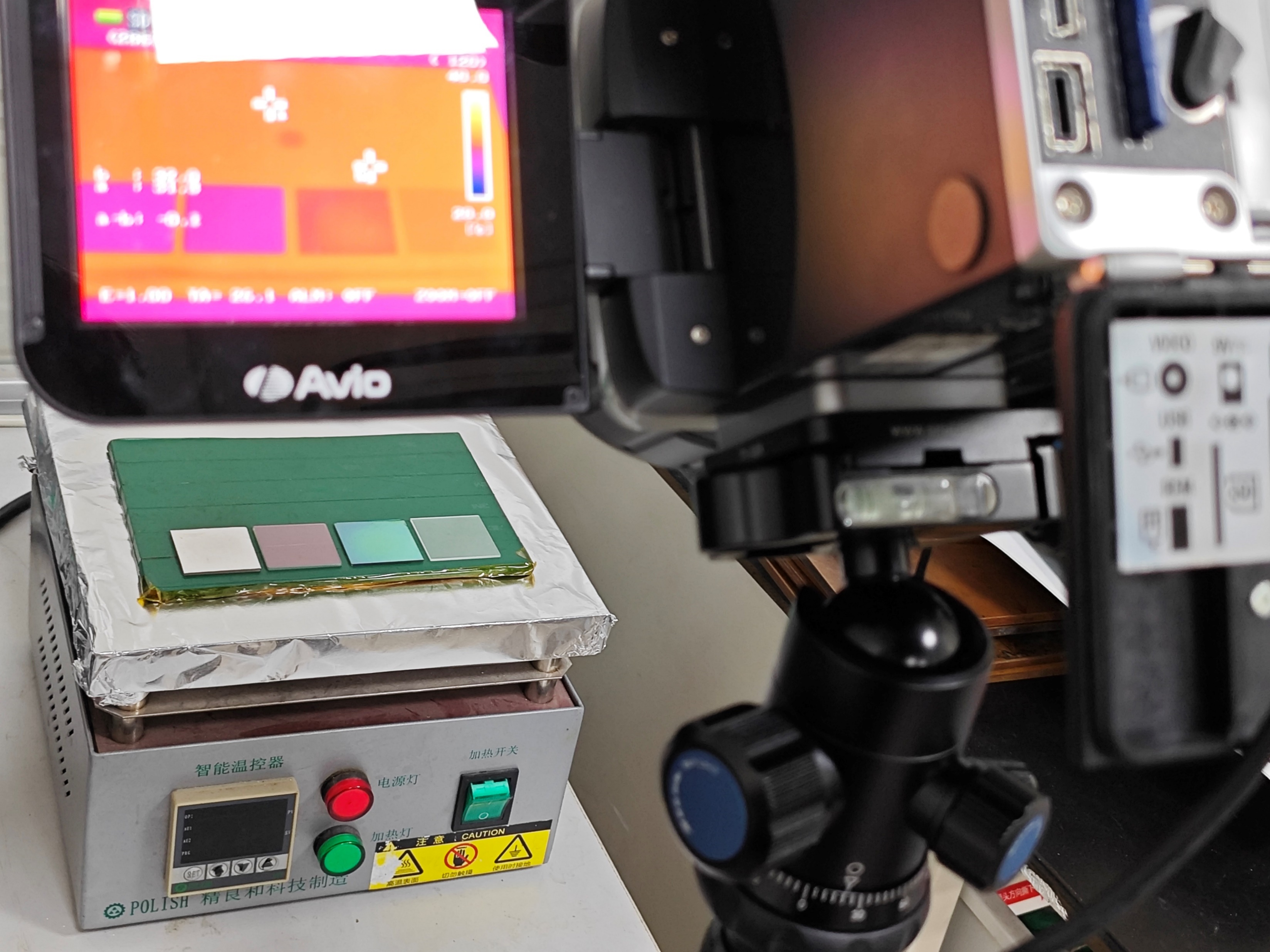


**Fig. S11 The photograph for infrared images shooting**.

**Fig. S12 Experimental setup for heating and cooling cycles testing.** The sample is heated by the infrared lamp and cooled in the natural environment with the ambient temperature around 20°C. The heating and cooling durations can be controlled via timer socket.

**Fig. S13 The experimental results of the visible spectral reflectance and corresponding reflective colors for flexible devices with various thickness combinations of the VO_2_ and HfO_2_ before and after the phase transition (a–e). (f) Photographs of flexible devices with different central wavelengths and states.** The designed central wavelength increases sequentially.

**Fig. S14 The reflectance spectra (a) and emissivity spectra (b) of the device prepared on the polyimide (PI) substrate.** The emissivity tunability can reach -0.48 in the atmospheric window.


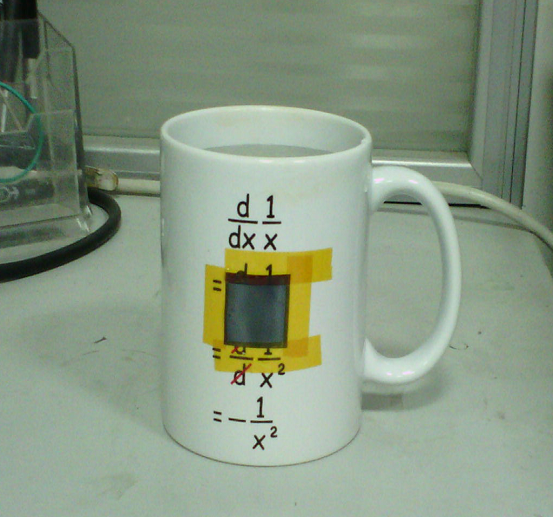


**Fig. S15 The photograph about the flexible device** **affixed to the cup.**

**Fig. S16 The photographs of folding tests setup (a) and** **the results of reflectance in 540 nm and emissivity tunability of the flexible sample (b).**

**Fig. S17 The photographs (a) and corresponding emissivity spectra (b) of the flexible device before and after the mechanical processing.** The scale bar shown in (a) represents 2 cm.

**Fig. S18** **Characterization for the flexible device.** The photographs (a) for the flexible sample with the sizes of 25×25 mm and 55×55 mm respectively. The XRD patterns (b) of PI substrate, VO_2_ on PI substrate and MSDR on PI substrate.

**Fig. S19 The photographs (a), corresponding infrared images (b) and the heat map of normalized radiation temperature deviation (c) of the large-scale flexible device.** The large-scale flexible device exhibits a remarkably uniform resistance distribution, particularly in the central region.

**Fig. S20 The measured surface temperature for device controlled by a voltage input (a) and corresponding photos for testing (b)**. The size of flexible MSDR device is 50×30 mm, with a 34.8 Ω conductive ITO film and a silver electrode deposited for Joule heating. The surface temperature is recorded by the thermocouple connected to a computer and a voltage of 2.5 V, 3 V and 3.5 V is applied to the device respectively.

**Fig. S21 The workflow of Neighboring Color Block Camouflage Algorithm.** The algorithm operates by analyzing adjacent regions in an image, ensuring that each color block seamlessly blends with its neighbors while maintaining irregular shapes and natural transitions. It reduces high-contrast edges by carefully selecting colors that are close in hue, saturation, or brightness, thereby enhancing the camouflage effect.

**Table 1 Summary of electrochromic devices with emissivity tunability gathered from the literature.**

| **Material** | **Device structure** | **Emissivity tunability** | **Stability** | **Ref.** |
| --- | --- | --- | --- | --- |
| Metal Oxide | ITO/WO_3_/Au | Δ*ε*_2.5–25 μm_=0.32 | Cycling numbers=10 | [^4^] |
|  | ITO/NiO/Ta_2_O_5_/Li/WO_3_/ITO/Ge/HfO_2_ | Δ*ε*_2.5–25 μm_=0.20 | Cycling time=10000s (coloring 30s, bleaching 30s) | [^5^] |
|  | golden metal grids/a-WO_3_/Ta_2_O_5_/a-WO_3_/golden metal grids | Δ*ε*_2–16 μm_=0.20 | Cycling numbers=300 | [^6^] |
|  | ITO/WO_3_/LiNbO_3_/NiO/Au | Δ*ε*_7–11 μm_=0.45 | Cycling numbers=1050 | [^7^] |
|  | ITO/WO_3_/LiTaO_3_/NiO_x_/ITO | Δ*ε*_8–14 μm_=0.37 | - | [^8^] |
|  | ITO/NiO/LiTaO_3_/Li_4_Ti_5_O_12_/WO_3_/ITO | Δ*ε*_2.5–25 μm_=0.34 | - | [^9^] |
|  | Li/WO_3_/ITO/Au/ITO/Nylon 66/Ta_2_O_5_/Li-based electrolyte/WO_3_/ITO | Δ*ε*_3–5 μm, 8–14 μm_ =0.76 | Cycling numbers>1000 | [^10^] |
|  | ITO/SiO_2_/ITO/Glass/ITO/NiO/ZrO_2_/Li/WO_3_/ITO | Δ*ε*_2.5–25 μm_=0.42 | Cycling time=30000s (each step for 30 s) | [^11^] |
|  | LTO nanoparticle | Δ*ε*_8–13 μm_=0.30 | Cycling numbers=100 | [^12^] |
| Conductive Polymer | Au grid/PANI-CAS/PAMPS/WO_3_/ITO | Δ*ε*=0.45 at 12 μm | Cycling numbers=900 | [^13^] |
|  | DBSA-doped PANI porous film | Δ*ε*_8–12 μm_=0.388 | - | [^14^] |
|  | CSA-doped PANI film | Δ*ε*_8–12 μm_=0.399 | Cycling numbers=100 | [^15^] |
|  | H_2_SO_4_-HClO_4_ co-doped PANI film | Δ*ε*_8–14 μm_=0.47 | Cycling numbers=200 | [^16^] |
|  | PANI film | Δ*ε*_2.5–25 μm_=0.391  Δ*ε*_2.5–25 μm_=0.383 | - | [^17^] |
|  | wrinkled PANI film/Au/Nylon 66/electrolyte/back electrode | Δ*ε*_8–14 μm_=0.375 | Cycling numbers=100 | [^18^] |
|  | electrode/PEDOT:Tos/electrolyte/PEDOT:Tos/electrode | Δ*ε*_8–14 μm_=0.153 | Cycling numbers=100 (significant degradation after 1000 cycles) | [^19^] |
| Graphene | single-walled carbon nanotubes based sandwich structure | Δ*ε*_8–14 μm_=0.55 | Cycling numbers=3500 | [^20^] |
|  | Nonvolatile ionic liquid-gated multilayer graphene | Δ*ε*_8–14 μm_=0.4 | 50% degeneration after the 780 cycles | [^21^] |
|  | ML-graphene/PE+IL/gold electrode | Δ*ε*=0.43 at 12 μm | Cycling numbers=500 | [^22^] |
|  | 12-layer graphene/ion gel/gold electrode | Δ*ε*_7.5–14 μm_=0.27 | Cycling numbers=500 | [^23^] |
|  | single-walled carbon nanotubes/ionic liquid/copper | Δ*ε*_7.5–14 μm_=0.50 | Cycling numbers=6000 | [^24^] |
| Metal | copper electrodeposition | Δ*ε*_7.5–13 μm_=0.85 | 21% degeneration after the 1800 cycles | [^25^] |
|  | silver electrodeposition | Δ*ε*_7.5–13 μm_=0.71 | Cycling numbers=350 | [^26^] |
| Quantum Well | GaAs/Al_0.3_Ga_0.7_As quantum well/2D photonic crystal plate | Δ*ε*=0.50 at the resonant wavelength | - | [^27^] |
|  | GaN/AlGaN Quantum-Well Photonic Crystal | Δ*ε*=0.15 at 4 μm | - | [^28^] |

-: The authors did not explicitly provide corresponding data in the manuscript.

**Table 2 Summary of VO_2_-based devices with structural color modulation gathered from the literature.**

| **Ref.** | **Device structure** | **Color modulation capability** | **Negative emissivity tunability** | **Materials impairing IR performance** |
| --- | --- | --- | --- | --- |
| [^29^] | Ag meta-surface/SiO_2_/VO_2_ | Yes | No | Ag |
| [^30^] | Ag meta-surface/Insulator/VO_2_/Ag | Yes | No | Ag |
| [^31^] | ITO/VO_2_/ITO/Ag | Yes | No | ITO, Ag |
| [^32^] | VO_2_/Au (self-organized  particles) | Yes | No | Au |
| [^33^] | Ag meta-surface/VO_2_/Ag | Yes | No | Ag |
| [^34^] | Ag/VO_2_/Al | Yes | No | Ag |
| [^35^] | TiO_2_/Ni/VO_2_/SiO_2_/Al | Yes | No | Ni, Al |
| [^36^] | VO_2_ meta-surface/Ag/VO_2_/Al | Yes | No | Ag, Al |
| [^37^] | MoS_2_ meta-surface/VO_2_/Au | Yes | No | Au |
| [^38^] | VO_2_/Al | Yes | No | Al |
| [^39^] | ZnS/Ge/VO_2_/Ag | Yes | No | Ag |
| [^40^] | VO_2_/PMMA/Au | Yes | No | Au |
| **This work** | **MSDR** | **Yes** | **Yes** | **-** |

**Table 3 Summary of VO_2_-based devices with negative emissivity tunability gathered from the literature.**

| **Ref.** | **Device structure** | **Emissivity in low temperature** | **Emissivity in high temperature** | **Emissivity tunability** | **Color modulation capability** |
| --- | --- | --- | --- | --- | --- |
| [^41^] | VO_2_/graphene/carbon nanotube | 0.86 | 0.49 | -0.37 | No |
| [^42^] | Al meta-surface/Al_2_O_3_/VO_2_/TiN | 0.81 | 0.4 | -0.41 | No |
| [^43^] | (Au/SU8) meta-surface/VO_2_/SiO_2_/Au | Not given | Not given | over 45% emissivity modulation in 7.5 μm | No |
| [^44^] | VO_2_ nanopowders | 0.82 | 0.36 | -0.46 | No |
| [^45^] | VO_2_/ZnS core-shell nanopowders | Not given | Not given | -0.28 | No (Only single gray-green) |
| [^46^] | VO_2_/quartz/silicone | 0.84 | 0.35 | -0.49 | No |
| [^47^] | VO_2_/carbon hybrid structure | 0.95 | 0.51 | -0.44 | No |
| [^48^] | W-doped VO_2_ nanopowders | Not given | Not given | -0.46 (undoped)  -0.30 (0.85% W-doped)  -0.28 (1.70% W-doped) | No |
| [^49^] | graded W-doped VO_2_ on sapphire | 0.75 | 0.35 | -0.40 | No |
| [^50^] | Al_2_O_3_/VO_2_/Al_2_O_3_/Al | 0.75 | 0.25 | -0.50 | No |
| [^51^] | Mo-doped VO_2_ nanopowders | 0.78 | 0.40 | -0.41^*^ | No |
| [^52^] | Al_2_O_3_/VO_2_/SiO_2_/Al | 0.75 | 0.21 | -0.52^*^ | No |
| [^53^] | VO_2_/HfO_2_/Al | 0.84 | 0.31 | -0.53 | No |
| [^54^] | VO_2_/mica/PMMA/Al | 0.82 | 0.35 | -0.50^*^ | No |
| [^55^] | Al_2_O_3_/W-doped VO_2_/SiO_2_/Al | 0.74 | 0.21 | -0.50^*^ | No |
| [^56^] | W/Al-doped VO_2_ nanopowders | 0.99 | 0.51 | -0.48 | No |
| [^57^] | W-doped VO_2_/KH-590 and NIPAAM modified PET | 0.95 | 0.68 | -0.27 | No |
| [^58^] | SiO_2_/W-SiO_2_/W/W-SiO_2_/W-doped VO_2_/Al_2_O_3_ | 0.81 | 0.45 | -0.46^*^ | No |
| **This work** | **MSDR** | **0.84** | **0.26** | **-0.58** | **Yes** |

^*^: Since the authors did not explicitly provide the emissivity tunability of the atmospheric window in the manuscript, this value was derived by extracting data from the corresponding emissivity spectrum and performing calculations.

**Table 4 The calculated thickness of the film for different central wavelength.**

| **Central wavelength (nm)** | **Thickness of VO_2_ (nm)** | **Thickness of HfO_2_ (nm)** |
| --- | --- | --- |
| 350 | 29.12 | 44.52 |
| 400 | 31.64 | 51.63 |
| 450 | 33.70 | 58.59 |
| 500 | 38.81 | 65.46 |
| 550 | 44.29 | 72.29 |
| 600 | 49.73 | 79.07 |
| 650 | 55.09 | 85.84 |
| 700 | 60.50 | 92.58 |
| 750 | 65.26 | 99.31 |
| 800 | 69.48 | 106.03 |

**Table 5 Summary of dynamic camouflage technology gathered from the literature.**

| **Ref.** | **Camouflage spectrum** | **Stimulation mode** | **Multi-scenario demonstration** | **Preparation simplification** | **Flexible** |
| --- | --- | --- | --- | --- | --- |
| [^59^] | MIR | Thermal | No | PLD, photolithography, PVD, CVD | No |
| [^43^] | MIR | Thermal | No | PVD, laser lithography | No |
| [^60^] | MIR | Mechanical | Yes | Chemical solvent preparation, PVD | Yes |
| [^61^] | MIR | Thermal | Yes | PVD | No |
| [^62^] | MIR | Electrical | Yes | Chemical solvent preparation | Yes |
| [^63^] | MIR | Thermal, electrical | Yes | PVD | No |
| [^41^] | MIR | Thermal, electrical | No | PVD, CVD | Yes |
| [^49^] | MIR | Thermal | Yes | PLD | Yes |
| [^64^] | Vis, MIR | Thermal | Yes | PVD | No |
| [^26^] | Vis, MIR | Electrical | Yes | PVD, Chemical solvent preparation | Yes |
| [^65^] | Vis, MIR | Electrical | Yes | Chemical solvent preparation | Yes |
| **This work** | **Vis, MIR** | **Thermal, electrical** | **Yes** | **PVD** | **Yes** |

**Reference**

1 Yariv, A. & Yeh, P. Electromagnetic propagation in periodic stratified media II Birefringence, phase matching, and x-ray lasers *. *Journal of the Optical Society of America* **67**,423, (1977).

2 Wang, D. *et al.* Structural color generation: from layered thin films to optical metasurfaces. *Nanophotonics* **12**, 1019–1081, (2023).

3 Wei, H. *et al.* Tunable VO_2_ cavity enables multispectral manipulation from visible to microwave frequencies. *Light Sci Appl* **13**, 54, (2024).

4 Li, Z. *et al.* Memristor of Tunable IR Emissivity Based on ITO/WO_3_/Au. *ACS Applied Nano Materials* **7**, 10625, (2024).

5 Sun, W. *et al.* Statically Multiple Colors and Dynamically Infrared Emissivity Modulation Compatible Electrochromic Devices via Simple Fabry–Perot Photonic Structures. *Laser & Photonics Reviews*, 2300476, (2023).

6 Chein Sheng Ly, K. *et al.* Visible‐to‐MIR broadband modulating electrochromic metal oxides‐based coating for thermal management. *Journal of the American Ceramic Society* **104**, 2143–2157, (2021).

7 Mei, Z. *et al.* A Colorful Electrochromic Infrared Emissivity Regulator for All-Season Intelligent Thermal Management in Buildings. *Adv Mater*, e2420578, (2025).

8 Zhang, X. *et al.* Preparation and performances of all-solid-state variable infrared emittance devices based on amorphous and crystalline WO_3_ electrochromic thin films. *Solar Energy Materials and Solar Cells* **200**, 109916, (2019).

9 Xiao, Y. *et al.* A visible-to-infrared broadband all-solid-state electrochromic device based Li_4_Ti_5_O_12_/WO_3_ for optical and thermal management. *Solar Energy Materials and Solar Cells* **268**, 112735, (2024).

10 Ding, Y. L. *et al.* Integrated Multispectral Modulator with Efficient Radiative Cooling for Innovative Thermal Camouflage. *Advanced Functional Materials*, 2500122, (2025).

11 Zhang, H. *et al.* All‐Solid‐State Transparent Variable Infrared Emissivity Devices for Multi‐Mode Smart Windows. *Advanced Functional Materials* **34**, 2307356, (2023).

12 Mandal, J. *et al.* Li_4_Ti_5_O_12_: A Visible‐to‐Infrared Broadband Electrochromic Material for Optical and Thermal Management. *Advanced Functional Materials* **28**, 1802180, (2018).

13 Topart, P. & Hourquebie, P. J. T. S. F. Infrared switching electroemissive devices based on highly conducting polymers. *Thin Solid Films* **352**, 243–248, (1999).

14 Tian, Y. *et al.* A comprehensive study of electrochromic device with variable infrared emissivity based on polyaniline conducting polymer. *Solar Energy Materials and Solar Cells* **170**, 120–126, (2017).

15 Zhang, L. *et al.* Fabrication of the infrared variable emissivity electrochromic film based on polyaniline conducting polymer. *Synthetic Metals* **248**, 88–93, (2019).

16 Xu, G. *et al.* Doping engineering of the flexible polyaniline electrochromic material through H_2_SO_4_–HClO_4_ multiple acids for the radiation regulation in snow environment. *Journal of Materials Chemistry C* **8**, 13336–13341, (2020).

17 Zhang, L. *et al.* Achieving variable infrared emissivity modulation regions of poly(aniline) films:the effect of film surface morphology on the optical tunability. *Dyes and Pigments* **187**, 109084, (2021).

18 Zhang, Y. *et al.* Rapid-response electrochromic devices with self-wrinkling polyaniline for enhanced infrared emissivity modulation. *Chemical Engineering Journal* **499**, 155960, (2024).

19 Brooke, R. *et al.* Infrared electrochromic conducting polymer devices. *Journal of Materials Chemistry C* **5**, 5824–5830, (2017).

20 Sun, Y. *et al.* Large‐Scale Multifunctional Carbon Nanotube Thin Film as Effective Mid‐Infrared Radiation Modulator with Long‐Term Stability. *Advanced Optical Materials* **9**, 2001216, (2020).

21 Sun, Y. *et al.* Flexible Mid-Infrared Radiation Modulator with Multilayer Graphene Thin Film by Ionic Liquid Gating. *ACS Appl Mater Interfaces* **11**, 13538–13544, (2019).

22 Salihoglu, O. *et al.* Graphene-Based Adaptive Thermal Camouflage. *Nano Lett* **18**, 4541–4548, (2018).

23 Lim, M. *et al.* Optically transparent and infrared tunable flexible camouflage device. *Nano Energy* **131**, 110189, (2024).

24 Ji, D., Li, X., Rezeq, M., Cantwell, W. & Zheng, L. Long-Term Stable Thermal Emission Modulator Based on Single-Walled Carbon Nanotubes. *ACS Appl Mater Interfaces* **15**, 37818–37827, (2023).

25 Sui, C. X. *et al.* Dynamic electrochromism for all-season radiative thermoregulation. *Nature Sustainability* **6**, 428–437, (2023).

26 Li, M., Liu, D., Cheng, H., Peng, L. & Zu, M. Manipulating metals for adaptive thermal camouflage. *Sci Adv* **6**, eaba3494, (2020).

27 Inoue, T., De Zoysa, M., Asano, T. & Noda, S. Realization of dynamic thermal emission control. *Nat Mater* **13**, 928–931, (2014).

28 Kang, D. D., Inoue, T., Asano, T. & Noda, S. Electrical Modulation of Narrowband GaN/AlGaN Quantum-Well Photonic Crystal Thermal Emitters in Mid-Wavelength Infrared. *ACS Photonics* **6**, 1565–1571, (2019).

29 Shu, F. Z. *et al.* Dynamic Plasmonic Color Generation Based on Phase Transition of Vanadium Dioxide. *Advanced Optical Materials* **6**, 1700939, (2018).

30 Song, S. *et al.* Tailoring active color rendering and multiband photodetection in a vanadium-dioxide-based metamaterial absorber. *Photonics Research* **6**, 492, (2018).

31 Wilson, K., Marocico, C. A. & Bradley, A. L. Dynamic structural colour using vanadium dioxide thin films. *Journal of Physics D: Applied Physics* **51**, 255101, (2018).

32 In, S. *et al.* Self‐Organized Gold Network–Vanadium Dioxide Hybrid Film for Dynamic Modulation of Visible‐to‐Near‐Infrared Light. *Advanced Photonics Research* **1**, 2000050, (2020).

33 He, J., Zhang, M., Shu, S., Yan, Y. & Wang, M. VO_2_ based dynamic tunable absorber and its application in switchable control and real-time color display in the visible region. *Opt Express* **28**, 37590–37599, (2020).

34 Zhao, J. *et al.* Flexible dynamic structural color based on an ultrathin asymmetric Fabry-Perot cavity with phase-change material for temperature perception. *Opt Express* **29**, 23273–23281, (2021).

35 Dai, P., Sun, K., Muskens, O. L., de Groot, C. H. & Huang, R. Inverse design of a vanadium dioxide based dynamic structural color via conditional generative adversarial networks. *Optical Materials Express* **12**, 3970, (2022).

36 Fang, J. *et al.* Thermally Tunable Structural Color Based on Patterned Ultra-Thin Asymmetric Fabry–Perot Cavity with Phase-Change Material. *Crystals* **13**, 996, (2023).

37 Cheng, T. *et al.* Dynamic tuning of optical absorbance and structural color of VO_2_-based metasurface. *Nanophotonics* **12**, 3121–3133, (2023).

38 Saini, S., P, A. & Verma, A. Dynamic multi-color switching using ultrathin vanadium oxide on aluminum-based asymmetric Fabry–Pérot resonant structure. *Applied Physics Letters* **124**, 011105, (2024).

39 Liang, S. *et al.* Structural color tunable intelligent mid-infrared thermal control emitter. *Ceramics International* **50**, 23611, (2024).

40 Zhang, X. *et al.* Tunable structural colors based on grayscale lithography and conformal coating of VO_2_. *Nanophotonics* **14**, 1123, (2025).

41 Xiao, L. *et al.* Fast Adaptive Thermal Camouflage Based on Flexible VO_2_/Graphene/CNT Thin Films. *Nano Lett* **15**, 8365–8370, (2015).

42 Yang, L. *et al.* Broadband thermal tunable infrared absorber based on the coupling between standing wave and magnetic resonance. *Optical Materials Express* **7**, 2767, (2017).

43 Chandra, S., Franklin, D., Cozart, J., Safaei, A. & Chanda, D. Adaptive Multispectral Infrared Camouflage. *ACS Photonics* **5**, 4513–4519, (2018).

44 Ji, H., Liu, D., Cheng, H., Zhang, C. & Yang, L. Vanadium dioxide nanopowders with tunable emissivity for adaptive infrared camouflage in both thermal atmospheric windows. *Solar Energy Materials and Solar Cells* **175**, 96–101, (2018).

45 Ji, H. N., Liu, D. Q., Zhang, C. Y. & Cheng, H. F. VO_2_/ZnS core-shell nanoparticle for the adaptive infrared camouflage application with modified color and enhanced oxidation resistance. *Solar Energy Materials and Solar Cells* **176**, 1–8, (2018).

46 Liu, D., Ji, H., Peng, R., Cheng, H. & Zhang, C. Infrared chameleon-like behavior from VO_2_(M) thin films prepared by transformation of metastable VO_2_(B) for adaptive camouflage in both thermal atmospheric windows. *Solar Energy Materials and Solar Cells* **185**, 210–217, (2018).

47 Wang, S. *et al.* Largely Lowered Transition Temperature of a VO_2_/Carbon Hybrid Phase Change Material with High Thermal Emissivity Switching Ability and Near Infrared Regulations. *Advanced Materials Interfaces* **5**, 1801063, (2018).

48 Ji, H., Liu, D. & Cheng, H. Infrared optical modulation characteristics of W-doped VO_2_(M) nanoparticles in the MWIR and LWIR regions. *Materials Science in Semiconductor Processing* **119**, 105141, (2020).

49 Tang, K. *et al.* A Thermal Radiation Modulation Platform by Emissivity Engineering with Graded Metal-Insulator Transition. *Adv Mater* **32**, e1907071, (2020).

50 Ao, X. *et al.* Self-adaptive integration of photothermal and radiative cooling for continuous energy harvesting from the sun and outer space. *Proc Natl Acad Sci U S A* **119**, e2120557119, (2022).

51 Chen, F. *et al.* Simultaneous tuning of the phase transition temperature and infrared optical properties of Mo-doped VO_2_ powders for intelligent infrared stealth materials. *Ceramics International* **49**, 25585–25593, (2023).

52 Liu, M. *et al.* Continuous Photothermal and Radiative Cooling Energy Harvesting by VO_2_ Smart Coatings with Switchable Broadband Infrared Emission. *ACS Nano* **17**, 9501–9509, (2023).

53 Gu, J. *et al.* Unprecedented Spatial Manipulation and Transformation of Dynamic Thermal Radiation Based on Vanadium Dioxide. *ACS Appl Mater Interfaces* **16**, 10352, (2024).

54 Tan, L. *et al.* Flexible composite film utilizing VO_2_ self-adaptive photothermal and infrared radiative cooling for continuous energy harvesting. *Optics Express* **32**, 22675, (2024).

55 Liu, M. *et al.* Sustainable All-Day Thermoelectric Power Generation From the Hot Sun and Cold Universe. *Small*, e2403020, (2024).

56 Li, M., Cheng, Y., Fang, C., Zhang, X. & Han, H. W/Al Co-doping VO_2_ nanoparticles for high performance passive infrared stealth films with enhanced durability. *Ceramics International* **50**, 1443–1451, (2024).

57 Wang, D. *et al.* Temperature response intelligent infrared stealth fabric based on the combined action of low infrared emissivity and heat isolation. *Journal of Alloys and Compounds* **1008**, 176735, (2024).

58 Wu, Z. *et al.* High-performance floating thermoelectric generator for all-day power supply. *Nano Energy* **133**, 110443, (2025).

59 Sun, R. *et al.* Broadband switching of mid-infrared atmospheric windows by VO_2_-based thermal emitter. *Opt Express* **27**, 11537–11546, (2019).

60 Zhang, Y. *et al.* Chameleon-inspired tunable multi-layered infrared-modulating system via stretchable liquid metal microdroplets in elastomer film. *Nat Commun* **15**, 5395, (2024).

61 Qu, Y. *et al.* Thermal camouflage based on the phase-changing material GST. *Light Sci Appl* **7**, 26, (2018).

62 Bo, Y. *et al.* Flexible active pixel-matrix ultrafast thermal camouflage using square-wave temperature. *Joule* **8**, 2160–2169, (2024).

63 Kim, C., Kim, Y. & Lee, M. Laser‐Induced Tuning and Spatial Control of the Emissivity of Phase‐Changing Ge_2_Sb_2_Te_5_ Emitter for Thermal Camouflage. *Advanced Materials Technologies* **7**, 2101349, (2022).

64 Jiao, S. *et al.* Metasurface with all-optical tunability for spatially-resolved and multilevel thermal radiation. *Nanophotonics* **13**, 1645–1655, (2024).

65 Wang, Y. *et al.* Visible-infrared compatible and independent camouflage with multicolor patterns and tunable emissivity. *Nanophotonics* **13**, 3123, (2024).
